# Metallocene Catalytic Insertion Polymerization of 1-Silene to Polycarbosilanes

**DOI:** 10.1038/srep16274

**Published:** 2015-11-06

**Authors:** Yuelong Tian, Min Ge, Weigang Zhang, Xiaoxu Lv, Shouquan Yu

**Affiliations:** 1 State Key Laboratory of Multi-phase Complex Systems, Institute of Process Engineering, Chinese Academy of Sciences, Beijing 100190, China; 2 University of Chinese Academy of Sciences, Beijing, 100049, China

## Abstract

Metallocene of zirconium were used as a catalyst for an insertion polymerization of 1-methylsilene directly into pre-ceramic precursor polyzirconocenecarbosilane (PZCS) during dechlorination of dichlorodimethylesilane by sodium, which exhibits high catalytic effectiveness with the maximum conversion ratio of polycarbosilane up to 91%. The average molecular weights of polymers synthesized are less than 1400, all with very narrow polymolecularities. The mechanism of catalytic polymerization was assumed to be similar to a coordination insertion polymerization of 1-olefins by metallocenes. The obtained PZCS show high ceramic yields with formation of composite ceramics of ZrC-SiC, which are novel polymeric precursors of ultra-high temperature ceramic (UHTC) fiber and composite.

Polycarbosilanes (PCS) are a family of polymers that contain both silicon and carbon atoms with or without an alternating order in their backbone. Thus, they are structural hybrids of polysilanes and polyolefines^1^,^2^,^3^,^4^. PCS have been synthesized mainly as industrial polymeric precursors for silicon carbide ceramic fiber or monoliths^1^,^5^. It is usually produced via thermal rearrangement of polymethylsilanes^6^,^7^ or by open ring polymerization of cyclic-carbosilane monomers^2^,^4^. Hyperbranched PCS was also reported by Interante and co-workers via monomolecular nonlinear step-growth polymerization and that these yield siliconcarbides of quite extraordinary properties^2^. The pathway using metallocenes of titanium(Ti), zirconium(Zr) and hafnium(Hf) catalytic polymerization of 1-olefin analogues (1-silene) as monomers for the production of PCS has not been considered. Although silene intermediates can be now prepared through many pathways such as thermal pyrolysis of cyclic carbosilanes or photo-decomposition of silylcarbenes, stable silene molecules are still difficult to produce because of their thermal lability^8^,^9^,^10^ and thus remain an active area of research. Interestingly, polycarbosilanes substituted with Ti, Zr, and Hf are in high demand for the production of SiC fibers and composites with thermal stability and oxidation resistance^11^,^12^,^13^,^14^,^15^ and dielectrical properties^16^.

Methylsilene (CH_2_=SiHMe) and dimethylsilylene (Me_2_Si:) are tautomeric intermediates achieved after a complete dechlorination of dimethyldichlorosilane or the photo/thermal decomposition of Me_2_Si(N_3_)_2_ and other precursors^8^,^9^. It is well known that transformation between these two transient intermediates depends strongly on the input energies and wavelength used. In general, methylsilene is a dominant moiety under visible light while dimethylsilylene is most common at 248 nm as shown by West or 254 nm described by Maier, respectively^8^,^9^.

Theoretically, a polycarbosilane with a repeated -CH_2_SiMeH- unit is clearly more thermodynamically stable than its isomer of polydimethylsilane (-SiMe_2_-). However, this was ignored after G Fritz’s systematic work that showed the thermal conversion of permethylsilanes into cyclic- and acyclic-carbosilanes^18^.

Here we describe the inhibition of dimethylsilylene polymerization for 1-methylsilene polymerization into polycarbosilanes via a one-pot reaction. This removes the requirements for an additional Kumada rearrangement via polydimethylsilanes (PDMS). We performed a catalytic insertion polymerization of methylsilene using zirconocene in toluene to yield a wine red polyzirconocenecarbosilane (PZCS). Based on the weight of the dimethyldichlorosilane reactant, the polymer product yield was about 91% which is much higher than Yajima’s process (<50%). Less than 10% of the polydimethylsilane was formed as insoluble byproducts that condensed with sodium chloride and residual sodium. We noted similar results when dichloromethylphenylsilane was used instead of dimethyldichlorosilane. In this case, the polycarbosilane is termed PZPCS for the purposes of this paper.

## Results and Discussion

**Figure 1:**
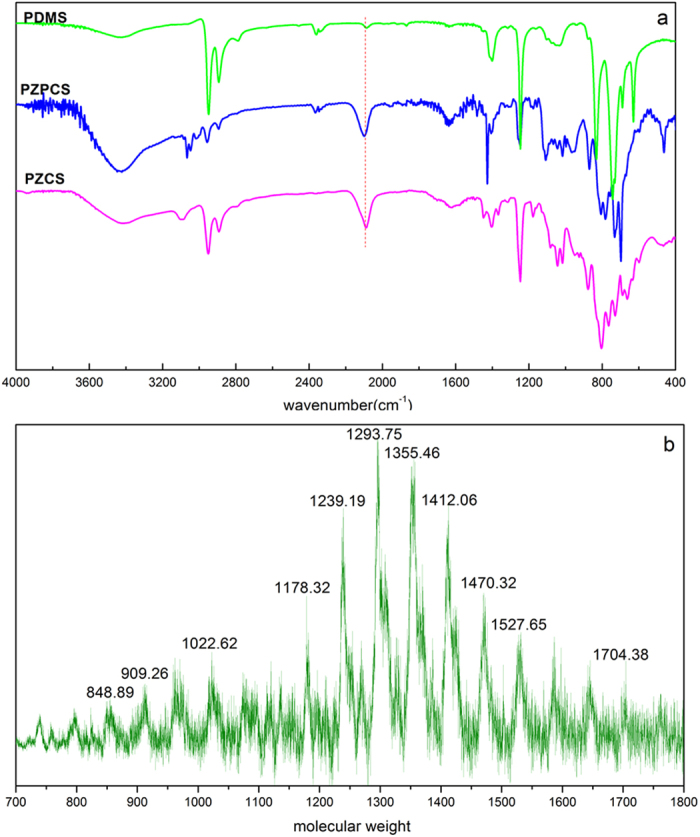
The FT-IR spectra of polydimethylsilane, polycarbosilane, and polyzirconocenecarbosilane (**a**), and the molecular weight and distribution of polyzirconocenecarbosilane by MALDI-TOF-MS (**b**).

Polycarbosilane with a repeating (-CH_2_-SiHR-) unit can be easily distinguished by its infrared spectrum. The FT-IR spectra of the three polymers (PDMS, PZCS and PZPCS) are shown in Fig. 1a. The Si-H bond and Si-CH_2_-Si bridge bond are generated from PZCS/PZPCS but are absent in PDMS. That is, the bands at 2,100 cm^−1^ (Si-H stretching), 880 cm^−1^ (Si-H deformation), 1020 cm^−1^ (Si-CH_2_-Si stretching) and 1,355 cm^−1^ (Si-CH_2_-Si bending) were observed in both PZCS and PZPCS, but not in PDMS.

Absorption bands at 600 to 920 cm^−1^ (Si-CH_3_ rocking and stretching), 1,250 cm^−1^ (Si-CH_3_ symmetric deformation), 1,400 cm^−1^ (Si-CH_3_ asymmetric deformation), 2,900 and 2950 cm^−1^ (C-H stretching of Si-CH_3_) were observed in all the three samples and confirm the presence of Si-CH_3_. Therefore, polycarbosilane was formed preferentially to PDMS when Cp_2_ZrCl_2_ was added to the Wurtz coupling reaction system. Otherwise, there are minor absorption bands in PZPCS at ~3,050 cm^−1^, 1,430 cm^−1^, 1,100 cm^−1^, 730 cm^−1^, and 700 cm^−1^. These are all from the phenyl groups.

The molecular weight and distribution of the synthesized PZCS were determined by a TOF-MS (time of flight mass spectrometer) and confirmed with GPC (gel permeation chromatography). The absolute weight values and molecular structure information can be analyzed directly from the TOF-MS data (Fig. 1b). The molecular weight average of PZCS is 1,300 with a maximum of 1,700 — this is similar to the PCS obtained by Yajima via a thermal rearrangement of PDMS^1^. Additionally, the difference between the adjacent peaks is around 58, which is the monomer molecular weight (-CH_2_SiMeH-). Therefore, the main degrees of polymerization are 20~25. The polymolecularities of PZCS obtained by GPC (ratio of M_w_ to M_n_) is 1.31, which is quite small for most polymers especially versus PC-470, which was synthesized by the thermal decomposition and condensation of PDMS in an autoclave at 470 °C for 14 h^5^,^17^ with average molecular weight 1750 and a very polymolecularity near 20. This is because PCS formed from decomposition of PDMS has a cross-linked molecular structure rather than linear chains.

**Figure 2:**
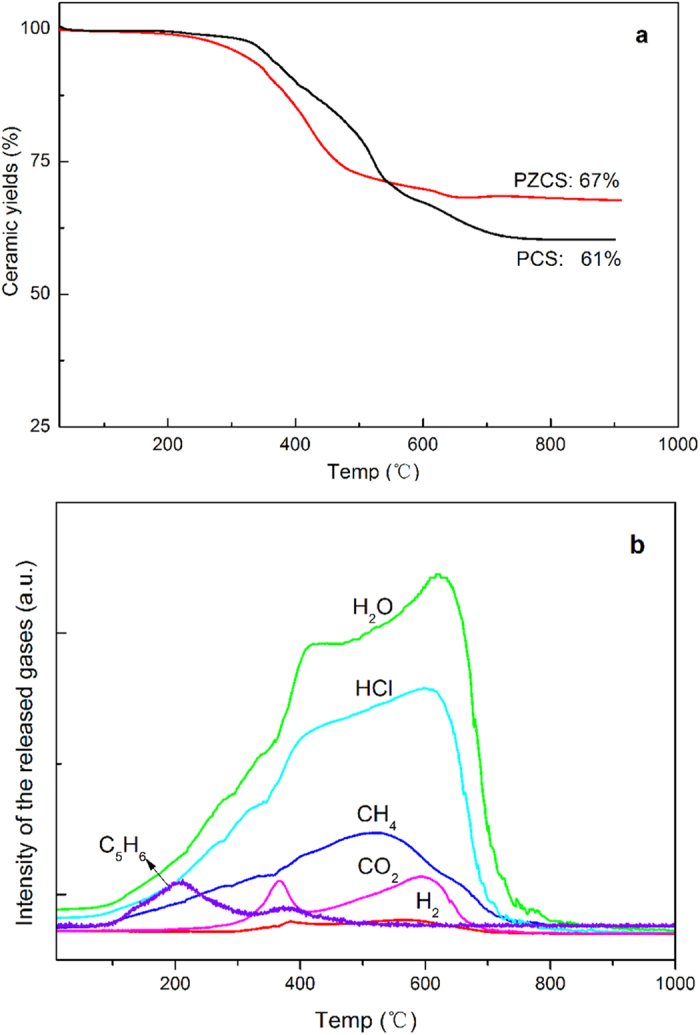
Thermal decomposition and ceramic yields of a polycarbosilane and polyzirconocenecarbosilane (PZCS) in argon (**a**) and mass spectrum determination of the released gaseous products during pyrolysis of PZCS (**b**).

Ceramic yields of the synthesized PZCS are quite an important characteristic because polycarbosilane is still widely used as pre-ceramic precursors. Thus, we used pyrolysis to study PZCS as well as commercially available PCS as a reference sample. This was done in argon up to 1,000 °C (Fig. 2a). The final yield of ceramic product with 15% ZrC is about 67%, which is higher than that of PCS with a value of 61%. On-line analysis of the gaseous products released during pyrolysis (Fig. 2b) indicates release of cyclopentadiene from metallocenes occurring from 150 °C to 300 °C with maximum release at 200 °C. Moreover, CH_4_, HCl, and H_2_ are the three major gaseous products with maxima at 520 °C, 580 °C, and 600 °C, respectively. Release of some CO_2_ and H_2_O can is seen at 370 °C and 620 °C. This is because of weak oxidation of polymers during processing.

**Figure 3:**
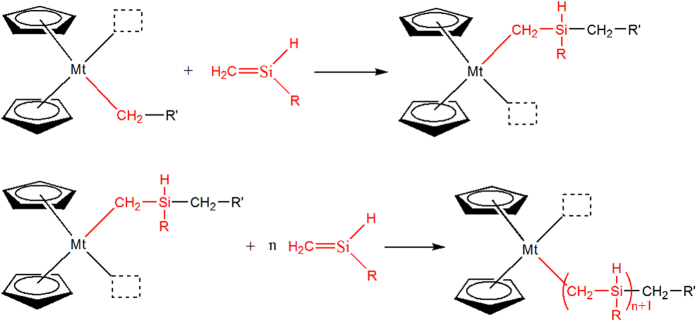
Metallocene catalytic insertion polymerization of 1-silene as analogues of 1-olefins. R = CH_3_, C_2_H_5_, C_6_H_5_, *etc*. R′ = H, Si(CH_3_)_2_Cl, Si(CH_3_)_2_-Si(CH_3_)_2_Cl, *etc*.

**Figure 4:**
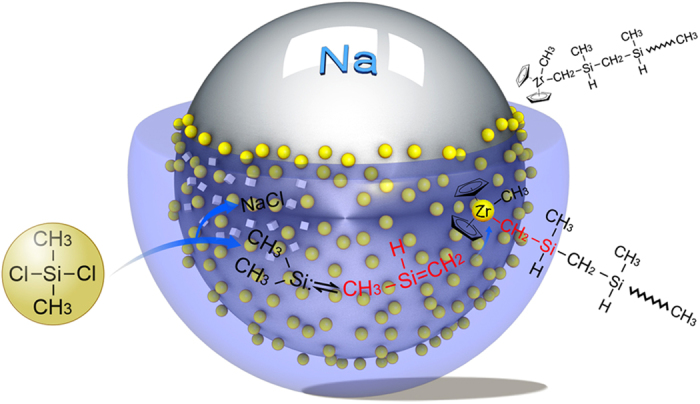
Schematic illustration of the surface-dominated metallocene catalytic polymerization of 1-methylsilene tautomeric intermediates.

The mechanism of catalytic polymerization of 1-silene tautomeric intermediates was assumed to be similar to those coordination insertion polymerizations of 1-olefin by metallocenes^19^,^20^,^21^. That is, the formation of an active center in these metallocenes by sodium similar to the role of methylalumoxanes (MAO) followed by adsorption of a silene monomer on this active center, insertion of the monomer into the Mt-C bond, and regeneration of an active center. In the process of metallocene initiated polymerization of olefin, these organometals are not split off from the reacting site after each reaction step as true catalysts are but rather stay at that site for many propagation steps like an initiator, which makes it possible for us to produce PZCS. These steps are summarized in Fig. 3 and schematic illustrated in Fig. 4.

Competition from dimethylsilylene polymerization is strongly inhibited simply because of a very low concentration of this moiety on the surface of the sodium. This is because the dechlorination reaction of dimethylchlorosilane is limited by diffusion and surface deactivation of sodium by metallocenes. This forms dimethylsilylenes that will be consumed as soon as possible by metallocene insertion. The molecular tautomeric moiety of the 1-methylsilane provides these metallocenes and offers high catalytic activity.

Dimethylsilylenes are highly reactive and thermal labile radicals that polymerize into cyclic or linear polysilanes^22^,^23^,^24^. Here, the dimerization from two silylenes into polysilane is overwhelmed by a one-by-one insertion growth into polycarbosilane that provides a formation rate of these tautomeric moieties that is lower than that of the metallocene catalytic insertion. This hypothesis is further confirmed by using some traditional Ziegler catalysts (TiCl_4_ and ZrCl_4_) with much lower catalytic activity. Neither offer polymerization of polycarbosilane, but rather produce polymethylsilane.

**Figure 5:**
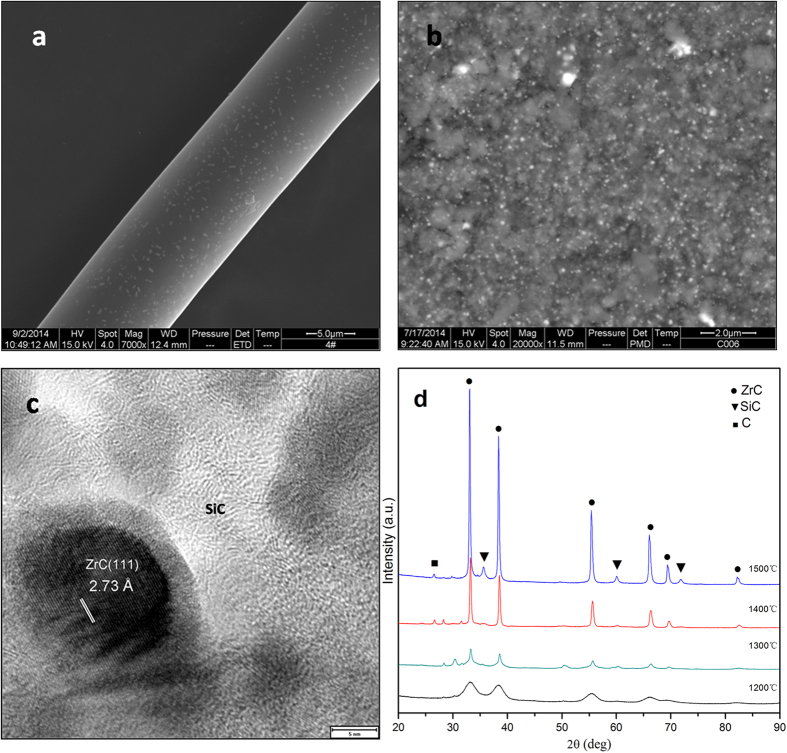
Ceramic fiber obtained from pyrolysis of the polyzirconocenecarbosilane and corresponding microstructure. (**a**) SEM morphology of the fiber; (**b**) cross-section SEM morphology of the fiber showing fairly homogeneous distribution of ZrC in SiC; (**c**) TEM showing nano-scale ZrC dispersed in a SiC matrix; and (**d**) XRD phases.

The synthesized PZCS has been used to produce composite ceramic fibers via a melting/spinning technique. Figure 5(a) shows the surface morphology of the PZCS-derived fibers pyrolyzed at 1,400 °C. These fibers are smooth and uniform with a mean diameter of 10 μm. Figure 5(b) shows the X-ray diffraction patterns of the fiber containing ZrC and SiC ceramic at a ZrC concentration of about 15% by mass. The HR-TEM micrograph of the ceramic fibers show that their crystalline phase consist of SiC and ZrC nanocrystallites, which are 5 to 30 nm in size with a fairly homogeneous distribution (Fig. 5c). Titanocene and hafnocene were also used for the catalytic polymerization of dichlorodimethylsilane with similar results for the polymer but with slightly lower yields of silicon conversion. The resulting polycarbosilane containing titanium and hafnium show excellent rheological characteristics for fiber melt spinning.

## Conclusions

In summary, direct polymerization of methylsilene into polycarbosilanes has been investigated using various metallocenes as catalyst during surface dechlorination of dichloromethylsilanes by sodium. For the first time, we show a metallocene catalytic insertion polymerization of tautomeric 1-silene into polycarbosilanes as analogues of polyolefins. The polycarbosilane synthesized through this molecular insertion process is suitable for spinning into MC-SiC composite ceramic fibers. These transition metal carbides act as reinforcements that improve the thermal and oxidation resistance of the SiC ceramic.

## Methods

### Synthesis

To prepare polycarbosilanes, we added 100 mL of toluene, 7.308 g of Cp_2_ZrCl_2_ and 4.6 g of metallic sodium into a 500 mL four-necked flask with a dropping funnel and reflux condenser. The mixture was then heated under a flowing nitrogen atmosphere to the toluene boiling point. After the sodium dissolved with magnetic stirring, 9.04 mL dimethyldichlorosilane (SiMe_2_Cl_2_) was added via the dropping funnel. The color of the solution quickly changed to wine red. The reaction completion was confirmed when the solution moved from acidic to neutral.

The precipitate was removed by suction filtration, and the polycarbosilane was separated from the solvent by rotary evaporation and termed polyzirconocarbosilane (PZCS). The product resulting from methylphenyldichlorosilane (SiMePhCl_2_) instead of dimethyldichlorosilane was named polyzirconophenylcarbosilane (PZPCS). The products were heated to 1,400 °C under Argon at 2 °C/min to pyrolize the products and make them ceramic.

The products were also spun into fibers under argon by heating them with spinning at 140 °C for 3 h. By raising the pressure, the melting products were extruded into green fibers. After curing, the green fibers were heated to 1,400 °C under an argon atmosphere at 2 °C/min and then held at 1,400 °C for 1 h.

### Characterization and measurements

Fourier transform infrared spectroscopy (FT-IR) was performed on a Thermo Nicolet 50 FT-IR from 4,000 to 400 cm^−1^. The molecular weight of the polymer was determined on a MALDI-TOF (Bruker Autoflex III). Gel permeation chromatography (GPC) analysis of the polymer was done on a Waters 1515 GPC instrument using toluene as the eluent and polystyrene as calibration standards.

Pyrolysis of the polymer was investigated with differential scanning calorimetry and thermal gravimetric analysis coupled with an online mass spectra analyzer (DSC–TG, Netzsch STA449C). This was operated under argon at a heating rate of 10 °C/min. The yield of ceramic was calculated as a ratio of the mass of products after heat treatment to the mass of polymer precursors. The ceramic phase compositions were identified by X-ray diffraction (XRD, Rigaku Dmax-rb) using CuKα radiation. The microstructures of the ceramics were characterized by scanning electron microscopy (SEM, S4800 Hitachi) and transmission electron microscopy (TEM, Tecnai G20) equipped with an X-ray energy dispersive spectrometer (EDS).

## Additional Information

**How to cite this article**: Tian, Y. *et al.* Metallocene Catalytic Insertion Polymerization of 1-Silene to Polycarbosilanes. *Sci. Rep.* **5**, 16274; doi: 10.1038/srep16274 (2015).
